# High-resolution mapping of heterochromatin redistribution in a *Drosophila *position-effect variegation model

**DOI:** 10.1186/1756-8935-2-1

**Published:** 2009-01-29

**Authors:** Maartje J Vogel, Ludo Pagie, Wendy Talhout, Marja Nieuwland, Ron M Kerkhoven, Bas van Steensel

**Affiliations:** 1 Division of Gene Regulation, Netherlands Cancer Institute, Plesmanlaan 121, 1066CX Amsterdam, The Netherlands; 2 Central Microarray Facility, Netherlands Cancer Institute, Plesmanlaan 121, 1066CX Amsterdam, The Netherlands

## Abstract

**Background:**

Position-effect variegation (PEV) is the stochastic transcriptional silencing of a gene positioned adjacent to heterochromatin. *white-mottled *X-chromosomal inversions in *Drosophila *are classic PEV models that show variegation of the eye color gene *white *due to its relocation next to pericentric heterochromatin. It has been suggested that in these models the spreading of heterochromatin across the rearrangement breakpoint causes the silencing of *white*. However, the extent of this spreading and the precise pattern of heterochromatin redistribution have remained unclear. To obtain insight into the mechanism of PEV, we constructed high-resolution binding maps of Heterochromatin Protein 1 (HP1) on *white-mottled *chromosomes.

**Results:**

We find that HP1 invades euchromatin across the inversion breakpoints over ~175 kb and ~30 kb, causing *de novo *association of HP1 with 20 genes. However, HP1 binding levels in these regions show substantial local variation, and *white *is the most strongly bound gene. Remarkably, *white *is also the only gene that is detectably repressed by heterochromatin. Furthermore, we find that HP1 binding to the invaded region is particularly sensitive to the dosage of the histone methyltransferase Su(var)3-9, indicating that the *de novo *formed heterochromatin is less stable than naturally occurring constitutive heterochromatin.

**Conclusion:**

Our molecular maps demonstrate that heterochromatin can invade a normally euchromatic region, yet the strength of HP1 binding and effects on gene expression are highly dependent on local context. Our data suggest that the *white *gene has an unusual intrinsic affinity for heterochromatin, which may cause this gene to be more sensitive to PEV than most other genes.

## Background

Position effect variegation (PEV) is the variation in expression of a gene caused by the stochastic inactivation of the gene in some cells of an otherwise homogeneous cell population. This variegation is often caused by the abnormal juxtaposition of the gene and a block of heterochromatin, which can be pericentric heterochromatin [1] or an array of inserted repeats that become heterochromatic [2]. PEV and related phenomena have been described in plants, yeasts and mammals ([3-6] and references therein). In *Drosophila*, PEV has been observed for a variety of genes (for an overview see [7]).

A prototypical PEV example involves the *Drosophila white *gene (*w*) that is normally located on the distal tip of the X chromosome. A chromosomal inversion named *white-mottled-4 *(*In(1)w*^*m4 *^or short *w*^*m4*^) places *white *next to the pericentric heterochromatin. Normally *white *is expressed in every ommatidium of the adult eye resulting in a red eye phenotype, but in *w*^*m4 *^mutants the eye contains patches of red and white tissue because the expression of *white *is variegating [8]. Different variants of *white-mottled *X chromosomes exist and are collectively referred to as *w*^*m*^. These variants have different inversion breakpoints and vary in the extent of mottling.

The chromosomal region that includes the white locus has a heterochromatin-like morphology in salivary glands of *w*^*m4*^larvae [9]. The silencing effect of PEV is generally attributed to a change in chromatin structure at the variegating locus that renders the gene less accessible to transcription factors [10]. In agreement with this, the *white *gene on *w*^*m *^is less accessible to its probe in *in situ *hybridization assays [9], and the chromatin of a reporter gene that is silenced by PEV often acquires distinct features such as a regular nucleosome array and insensitivity to nucleases [11-13].

A set of specialized proteins, collectively termed heterochromatin proteins, mediate the structural changes seen at variegating loci. Heterochromatin Protein 1 (HP1) is widely accepted as a defining marker of heterochromatin in most eukaryotes, and is also one of the most studied components of heterochromatin [14,15]. It is abundantly associated with pericentric heterochromatin [16], but also found at hundreds of genes dispersed along the chromosome arms in fly and human cells [17-21]. Mutations in HP1 strongly suppress the silencing of reporter genes in *w*^*m *^and other PEV models [16,22,23], indicating that HP1 is an essential component of heterochromatin.

HP1 contains two conserved protein domains, the N-terminal chromodomain and the C-terminal chromoshadow-domain. The chromodomain of HP1 recognizes di- and trimethylated lysine 9 of Histone H3 (H3K9me2/3) [24-26]. Su(var)3-9 is a key histone methyltransferase responsible for H3K9 di- and trimethylation [25,27,28]. The chromoshadow domain of HP1 can also bind Su(var)3-9 directly [29,30]. Molecular mapping in *Drosophila *cells has shown that HP1 and Su(var)3-9 colocalize at most of their target loci [18]. The localization of HP1 to heterochromatic regions [30] and genes [18] depends on Su(var)3-9, except on *chr 4 *where HP1 localization depends on *Drosophila *SETDB1 [31,32].

Several models have been proposed to explain the molecular mechanism of PEV (for an overview see [6]). A requirement for such a model is that it must explain how a gene (such as *white *in the case of *w*^*m4*^) is silenced by heterochromatin even though it can be located hundreds of kb away from the heterochromatin-euchromatin junction (see [7] and references therein).

A popular model involves the linear propagation of heterochromatin protein complexes *in cis *along the chromatin fiber ('oozing model'). This would cause a contiguous stretch of originally euchromatic DNA to become invaded by heterochromatin. The endpoint of the new heterochromatin domain may vary between cells, which could account for the variegating silencing. The interactions between H3K9me2/3, HP1 and Su(var)3-9 suggest a model for the mechanism by which heterochromatin could be propagated *in cis *along the chromatin fiber: When HP1 binds to H3K9me2/3, it recruits Su(var)3-9, which can methylate H3K9 on neighboring histones, which in turn will recruit more HP1 [24,25]. This mechanism is supported by observations in *Schizosaccharomyces pombe *suggesting that the Su(var)3-9 homolog Clr4 initially methylates H3K9 independent of the HP1 ortholog Swi6, whereas subsequent maintenance and spreading of H3K9 methylation is Swi6 dependent [33].

Some observations in *Drosophila *challenge the linear propagation model of PEV. Certain genes appear to 'escape' silencing inside a presumed heterochromatic region. For example, close examination of two reporter genes in the *w*^*m *^variant *w*^*mMc *^[34] indicated that the *roughest *gene, which is farthest from the heterochromatin-euchromatin junction, can be inactive in some cells where *white*, which is closer to the junction, is active. These and other analyses of reporter genes in a fly PEV model [35,36] argue against linear propagation of silencing.

A second model proposes that the formation of a heterochromatin domain may occur in a discontinuous ('hopping') fashion, leaving certain genes in a euchromatic state. This discontinuous binding is explained by local differences in the binding affinity, determined by DNA sequence or epigenetic marks. In support of this, morphologically discontinuous heterochromatin has been observed on polytene chromosomes in regions undergoing PEV [37]. This could explain why silencing can 'skip' some genes. Finally, looping of the chromatin fiber may bring certain genes into contact with a block of pericentric heterochromatin, leading to silencing [38-40], while some genes in the intervening region may remain unaffected [6].

In order to understand the mechanism of PEV and to discriminate between the different models, it is essential to know the precise distribution of heterochromatin along the chromosomal region around the chromosomal rearrangement breakpoints. Here, we report the high-resolution mapping of HP1 in *Drosophila *along wild-type and *w*^*m *^chromosomes. The results reveal that HP1 encroaches into nearly 200 kb of normally euchromatic DNA in *w*^*m*^. However, the level of HP1 binding shows substantial local variation along this region, and we find that the majority of genes in this region are not silenced by the invading heterochromatin, possibly because the local binding levels of HP1 are not high enough for effective repression.

## Results

### The binding pattern of HP1 on wild-type, *w*^*m4e*^, and *w*^*m51b *^X chromosomes

We employed the DamID technique [41] to study the redistribution of HP1 on *w*^*m *^chromosomes. DamID was previously used to identify the natural binding sites of *Drosophila *HP1 in Kc cells [17,18] and in whole adult flies [42]. These studies found a strong enrichment of HP1 in pericentric regions and on chromosome 4, in agreement with immunofluorescence microscopy data [18]. DamID maps of HP1 showed a strong overlap with several other heterochromatin proteins but not with euchromatin proteins [17,18,43]. DamID also confirmed earlier microscopy observations [30], indicating that the binding of HP1 to chromosome 4 is less dependent on the presence of Su(var)3-9 than binding of HP1 to all other chromosomes [18]. These earlier studies thus validated the use of DamID to map HP1 binding.

**Figure 1:**
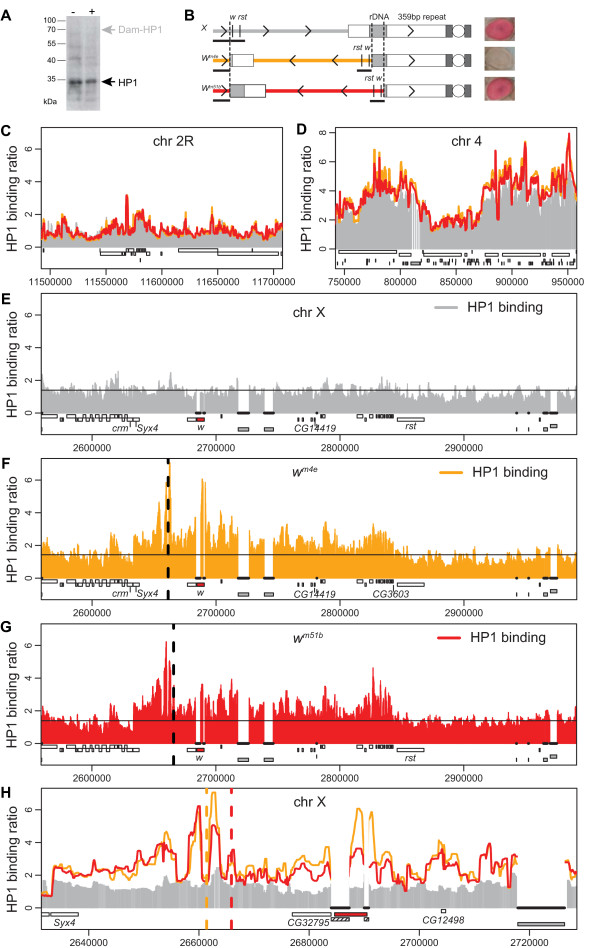
**HP1 binding maps**. A) Dam-HP1 fusion protein is expressed at very low levels. Western blot is shown of fly head protein extracts of *w*^*1118 *^controls (-) or a transgenic line expressing the Dam-HP1 fusion protein from the uninduced *hsp70 *core promoter (+), probed with an anti-HP1 antibody [16]. Position of HP1 and expected position of (non-detectable) Dam-HP1 fusion protein are marked by arrowheads. B) Cartoon showing the wild-type *chr X *and the inverted X chromosomes (*w*^*m4e *^and *w*^*m51b*^) used in this study. Inversion breakpoints are indicated with dashed lines. *w *= *white*, *rst *= *roughest*. Arrow heads (>) indicate the direction on the wild-type *chr X*. Black-underlined regions are represented on our microarray. Pictures on the right of each chromosome show eye color of representative males. C-D) Chromosomal maps of HP1 binding to ~200 kb regions of *chr 2R *(C) and *chr 4 *(D). Grey sticks show wild-type Dam-HP1/Dam binding ratio. Orange and red lines indicate Dam-HP1/Dam binding ratio in *w*^*m4e *^and *w*^*m51b*^, respectively. A running median filter (window size 5) was applied to suppress noise. Genes are plotted as white rectangles. Transposable elements (TEs) are plotted below the genes as grey rectangles. E) Chromosomal map of HP1 binding to the wild-type (*Oregon-R-S*) *chr X *for the ~400 kb region surrounding the *white *gene. Black horizontal line indicates the average Dam-HP1/Dam binding ratio on the first 3.2 Mb of *chr X*. TE and *mini-white *sequences possibly cross-hybridize on the microarray and are therefore masked in the plots (indicated with black, uninterrupted thick lines). *white *is depicted as a red rectangle, *crm = cramped*, *Syx4 = Syntaxin 4*. Other features are the same as in C-D. F-G) Chromosomal map of HP1 binding to *w*^*m4e *^(F), and *w*^*m51b *^(G). Vertical dashed line indicates euchromatic inversion breakpoint. In reality the sequence on the right of the break is attached to the pericentric heterochromatin. H) Close-up of the ~90 kb surrounding the *white *gene (red rectangle). *mini-white *sequences (hatched rectangles), which are present in the DamID expression vector, possibly cross-hybridize to the endogenous *white *sequence and are therefore masked. Colors as in E-G.

We expressed the Dam-HP1 fusion or unfused Dam from the *hsp70 *core promoter [42], which at 25°C drives only extremely low expression levels, as confirmed by the lack of a detectable band on a Western blot (Figure 1A). This trace amount of Dam-HP1 expression relative to endogenous HP1 is not only a requirement for DamID [41] but it also ensures that the Dam-HP1 fusion protein does not significantly alter heterochromatin by itself. In order to map HP1 binding on different variants of *w*^*m *^inverted X chromosomes we downscaled DamID for use in adult fly heads. We used whole heads because chromosomal conformations correlating with an eye phenotype can be readily observed in the central nervous system [38,39]. We examined two different chromosomal inversions, *w*^*m4e *^and *w*^*m51b*^, which display a different eye phenotype but have a very similar chromosomal structure. *w*^*m4e *^males have almost completely white eyes with only a few red patches, whereas the eyes of *w*^*m51b *^males are red and resemble wild-type eyes (Figure 1B). The euchromatic breakpoints of the *w*^*m4e *^and *w*^*m51b *^inversions are located ~29 and ~25 kb downstream of the transcriptional start of the *white *gene (see Methods and [34]), which is normally located on the distal tip of the chromosome. The heterochromatic breaks are proximal and distal to the rDNA, in heterochromatin block h28 and h30, respectively [44,45] (Figure 1B). As a control we mapped HP1 on the wild-type X chromosome in the *Oregon-R-S *strain.

To identify the DNA from the chromatin that was bound by HP1 *in vivo*, we designed a high-density 44 K oligonucleotide microarray with 60-mer probes corresponding to unique sequences from the first 3.2 Mb of the X chromosome (chr). As positive control regions we included parts of the genome where HP1 levels are expected to be high, i.e., the centromere-proximal 0.75 Mb of *chr 2R*, and the complete mostly heterochromatic *chr 4 *[18,42]. As a negative control region we included a euchromatic 0.5 Mb segment of *chr 2R *(position 11.4–11.9 Mb), where HP1 binding is restricted to a small number of genes [18,42]. We normalized the log_2 _binding ratios to the average of this euchromatic *chr 2R *segment (see Methods). Therefore, positive log_2 _binding ratios (i.e. ratios > 1) can be interpreted as more HP1 binding than found on average in euchromatin.

To visualize the binding of HP1 in adult male heads we made chromosomal maps (Figure 1C–H). In agreement with previous observations [18] we detect high HP1 levels on the centromere-proximal 0.75 Mb part of 2R (data not shown) and along most parts of *chr 4 *(Figure 1D, and data not shown). Next, we focused on the HP1 binding pattern in a 400 kb region surrounding the *white *gene (Figure 1E–H). Compared with *Oregon-R-S *(Figure 1E), HP1 levels on *w*^*m4e *^(Figure 1F) and *w*^*m51b *^(Figure 1G) are clearly elevated in the regions next to the inversion breakpoint. Effects of the inversion are seen on both sides of the euchromatic inversion breakpoint (vertical dotted lines in Figure 1F–H). HP1 levels are elevated over a ~175 kb region stretching from the break towards *CG3603*, the gene upstream of *roughest (rst)*, and over a ~30 kb region downstream from the break including *Syx4*, but clearly not *crm *(Figure 1H). We will refer to this ~200 kb region of the *chr X *surrounding the euchromatic inversion breakpoint as X_Syx4-CG3603_.

On the wild-type *chr X *the region corresponding to X_Syx4-CG3603 _shows no prominent HP1 binding (comparable to the average of the entire X chr), indicating that this region does not possess an intrinsic bias for HP1. In contrast, on *w*^*m4e *^and *w*^*m51b *^almost all microarray probes in this region report elevated levels of HP1. Thus, the inversion induces HP1 association with the entire X_Syx4-CG3603 _region, although gaps smaller than the estimated resolution of DamID (about 1–2 kb [41]) cannot be ruled out. Despite this apparent contiguous HP1 binding, reproducible local variations in the HP1 DamID signal were observed, with the highest levels of HP1 close to the inversion breakpoint and on the *white *gene. Overall, the HP1 binding ratios in X_Syx4-CG3603 _are somewhat lower than those in the pericentric heterochromatic regions of *chr 4 *and *chr 2R *(*cf*. Figure 1D and data not shown). Taken together, these data suggest that *w*^*m *^chromosomal inversions induce invasion of HP1 from heterochromatin into the neighboring euchromatic regions. HP1 binds to these regions in a contiguous fashion, but the binding levels show substantial local variation.

**Figure 2:**
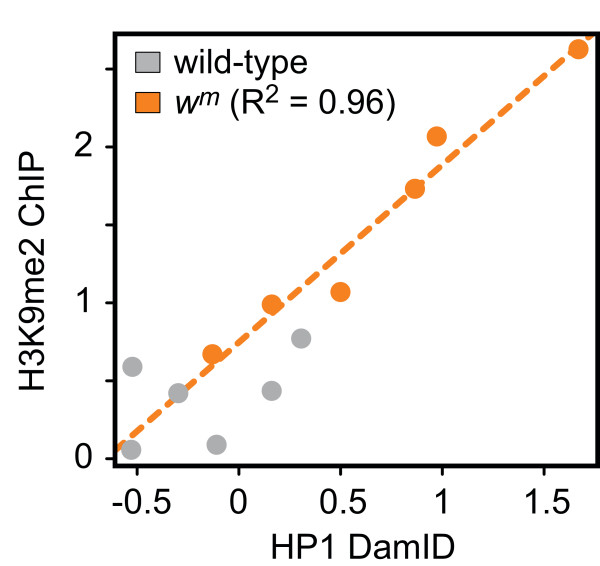
**Similarity of HP1 and H3K9me2 profiles**. Scatter plot comparing the HP1 DamID data with previously published [46] H3K9me2 ChIP data for six loci in the region probed in Figure 1, E-G. HP1 values represent the average of tiling array probes located within 500 bp of the sequences probed in [46]. Grey dots = wild-type; orange dots = *w*^*m4e*^. Orange dotted line represents a linear regression fit to the data.

These HP1 data are consistent with a previous ChIP analysis of a *w*^*m *^chromosomal inversion, which detected H3K9me2 in the region from *white *to *rst*, with the highest levels on *white *[46]. Direct comparison reveals that, at the six loci in this region for which H3K9me2 levels are known, an excellent correlation (*R*^2 ^= 0.96) exists between our HP1 DamID signals and the H3K9me2 ChIP data (Figure 2). From this we conclude that our HP1 maps are of high quality.

### Redistribution of HP1 is mostly restricted to X_Syx4-CG3603_

**Figure 3:**
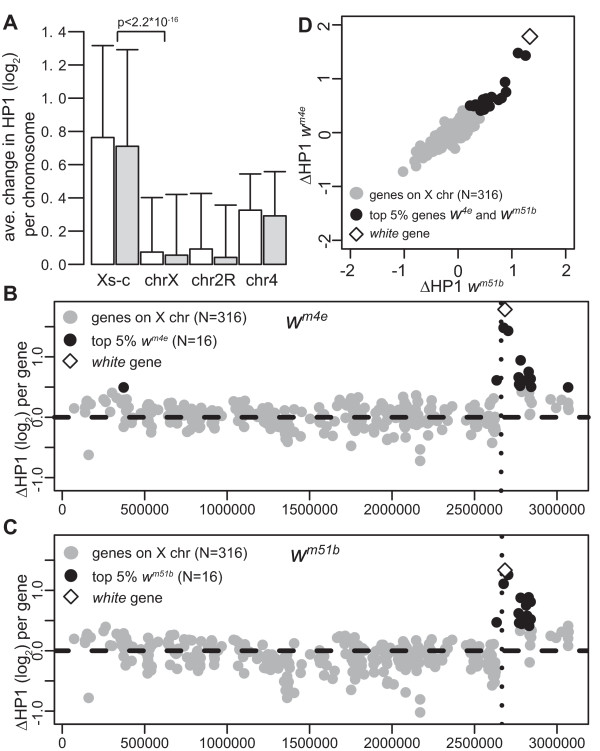
**Effects of *w*^*m *^inversions are found locally on X**. A) Barplot showing the difference in HP1 binding for each probe on the array averaged per chr. Open bars show difference in binding between *w*^*m4e *^and wild-type, grey bars between *w*^*m51b *^and wild-type. Error bars indicate standard deviation of differences. Xs-c = X_Syx4-CG3603_; chrX = first 3.2 Mb of *chr X *excluding X_Syx4-CG3603_; chr2R = regions of *chr 2R *that are covered on the microarray; chr4 = *chr 4*. *P *value from Wilcoxon rank sum test. B-C) Chromosomal maps of first 3.4 Mb of X chr, with ΔHP1 (average change in log_2 _HP1 binding ratio) per gene. ΔHP1 between *w*^*m4e *^and *Oregon-R-S *(B), ΔHP1 between *w*^*m51b *^and *Oregons-R-S *(C). Black dots show top 5% of genes (*n *= 16) for which ΔHP1 is largest. Inversion breakpoints are indicated with black dotted lines. D) Bivariate scatterplot of data presented in B and C.

The HP1 pattern on the X chromosome outside X_Syx4-CG3603 _looks highly similar in all genotypes examined (Figure 1D–F, data not shown), suggesting that the *w*^*m *^chromosomal inversions affect HP1 binding patterns only locally. To examine this in more detail we calculated the average change in HP1 binding ratios for each of the probed chromosomal segments (Figure 3A). HP1 binding levels in X_Syx4-CG3603 _are 1.6–1.7-fold higher on *w*^*m4e *^and *w*^*m51b *^compared with the wild-type X chr, whereas the remainder of the *chr X *and pericentric heterochromatin on *chr 2R *were unaltered (< 1.07-fold change). We also noticed a smaller increase (~1.2-fold) in HP1 binding ratios on *chr 4*, which might be a secondary effect of the X chromosomal inversion, or due to other differences in the genetic background. In any case, the most pronounced effects of the *w*^*m *^inversions are found locally in X_Syx4-CG3603_.

In some rearrangements that give rise to PEV, genes up to ~2 Mb from the breakpoint have been shown to variegate [47]. To systematically identify the genes on *chr X *where HP1 binding was affected most, we calculated the average change in HP1 binding log-ratio (ΔHP1) for each gene on *w*^*m4e *^and *w*^*m51b *^chromosomes relative to the wild-type *chr X *(Figure 3B–C). We then selected the top 5% of genes (*n *= 16) for which ΔHP1 is largest in *w*^*m4e *^(Figure 3B) or *w*^*m51b *^(Figure 3C). Twelve out of 16 affected genes overlap between *w*^*m4e *^and *w*^*m51b *^(Figure 3D), and these genes are all located in the X_Syx4-CG3603 _region. This result reinforces the notion that effects of the chromosomal inversion on HP1 patterns are mainly found locally.

### Increased HP1 binding in X_Syx4-CG3603 _primarily affects white expression

**Figure 4:**
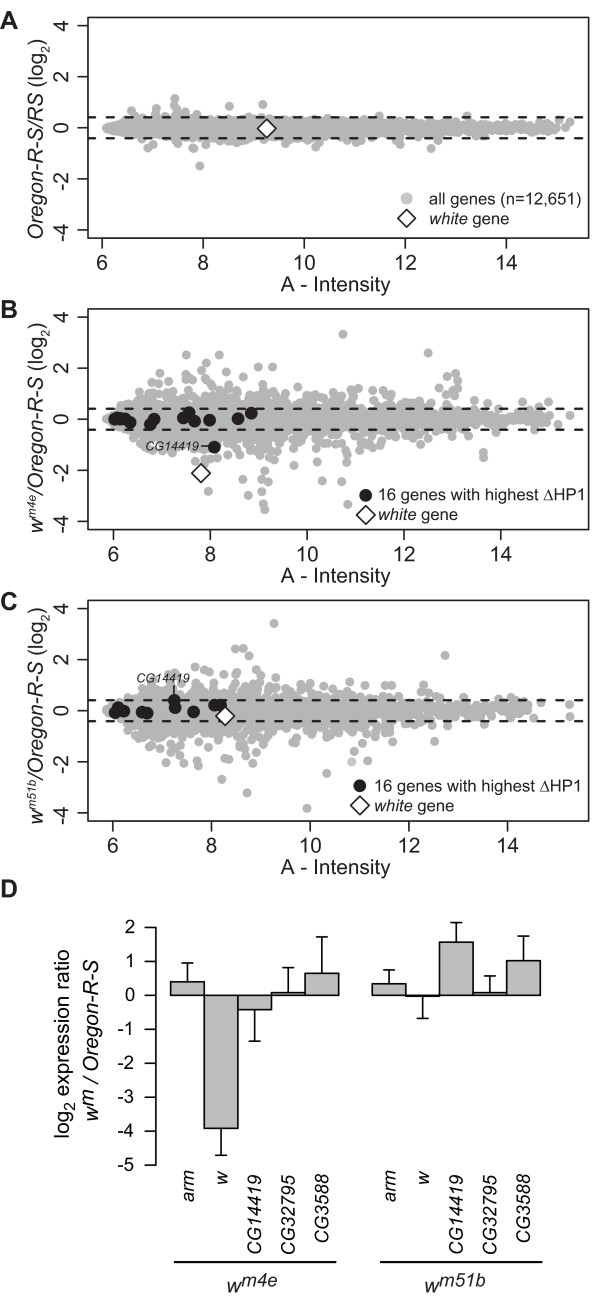
**Expression profiling shows that *white *is selectively down-regulated in *w*^*m4e*^**. A-C) MA plots of expression profiles of log_2 _*Oregon-R-S *Self-self (A), log_2 _(*w*^*m4e*^/*Oregon-R-S*) (B), and log_2 _(*w*^*m51b*^/*Oregon-R-S*) (C), respectively. In these plots, for each gene, change in expression (log_2_) is plotted against the expression level. A = average fluorescence intensity (log_2 _√(Cy5xCy3)). Dashed lines indicate four times the standard deviation. D) qRT-PCR measurements of changes in gene expression in *w*^*m *^mutants versus wild-type (*Oregon-R-S*). Bars show average log_2 _ratios of five replicate experiments. Error bars represent standard deviations. Expression levels were normalized to the housekeeping gene *Ide*, which is located on chr 3L; *arm *is located outside of X_Syx4-CG3603 _and serves as an additional control.

The *white *gene is the gene that shows the strongest increase in HP1 levels, both on the *w*^*m4e *^and on the *w*^*m51b *^chr. Nevertheless, the eye color phenotype of these two lines is remarkably different: *w*^*m4e *^males have almost white eyes, whereas the eye color of *w*^*m51b *^males is virtually wild type (Figure 1B). From the eye color phenotypes we would predict that the *white *gene is down regulated in the *w*^*m4e *^line and normally expressed in the *w*^*m51b *^line. To investigate the transcriptional status of *white *and other genes, we generated microarray expression profiles of male heads from *w*^*m4e *^and *w*^*m51b *^and control *Oregon-R-S *flies. The MA-plot is a graphical way to visualize expression levels (fluorescence intensity) and change in expression levels (log-ratios) at the same time, that can be used to identify differentially expressed genes. The MA-plot of a set of *Oregon-R-S *self-self hybridizations (Figure 4A) was used to estimate the biological and technical noise. MA-plots of *w*^*m4e *^against *Oregon-R-S *and *w*^*m51b *^against *Oregon-R-S *are shown in Figure 4B and 4C, respectively. First, we focused on the 20 genes in X_Syx4-CG3603 _(Table 1). More than half of these genes had an expression level that was too low to allow detection of differential expression on our microarray platform (Table 1, genes with A < 7, stringent and arbitrary cutoff). These genes are in the left part of the A-axis in Figure 4B and 4C. Eight out of 20 genes were expressed at sufficient levels (*A *> 7) to detect their possible differential expression. As expected, *white *was down-regulated in *w*^*m4e *^(log_2 _ratio = -2.11, *P *< 10^-45^, Figure 4B and Table 1) but not in *w*^*m51b *^(log_2 _ratio = -0.21, *P *= 0.18, Figure 4C). Other than *white *only one out of the eight genes (*CG14419*) showed a modest down-regulation (log_2 _ratio = -1.10) in *w*^*m4e *^compared with *Oregon-R-S*, while none of the other genes displayed a detectable reduction in their expression. In *w*^*m51b *^none of the eight genes was more than about 30% up- or down-regulated compared with *Oregon-R-S *(Table 1).

**Table 1:** Microarray expression profiling.

					*w*^*m4e*^/***Oregon-R-S***			*w*^*m51b*^/***Oregon-R-S***		
**CG**	**FBgn**	**Gene symbol**	**Start (on *chr X*)**	**End (on *chr X*)**	**M (log_2 _ratio)**	**A**	**P**	**M (log_2 _ratio)**	**A**	***P*-value**

CG13373	FBgn0029522		371554	372216	**-0.20**	**6.76**	**4.79E-01**	0.02	6.69	9.30E-01
CG2715	FBgn0024980	Syx4	2633120	2638099	**0.25**	**7.56**	**2.04E-04**	**0.12**	**7.26**	**9.16E-03**
CG32795	FBgn0040384		2676939	2683975	**-0.04**	**7.98**	**5.46E-01**	**-0.05**	**7.64**	**4.91E-01**
CG2759	FBgn0003996	w	2684632	2690499	**-2.11**	**7.81**	**< 10E-45**	**-0.21**	**8.28**	**1.83E-01**
CG12498	FBgn0040356		2704000	2704785	**0.03**	**6.14**	**5.43E-01**	**0.01**	**6.16**	**7.97E-01**
CG14416	FBgn0040352		2766023	2766854	**0.00**	**6.02**	**9.59E-01**	**-0.08**	**6.04**	**2.69E-01**
CG14417	FBgn0040353		2769564	2770556	**0.05**	**6.07**	**6.13E-01**	**0.05**	**6.13**	**6.24E-01**
CG14418	FBgn0040354		2776599	2777666	**0.01**	**6.24**	**9.31E-01**	0.01	6.20	9.03E-01
CG14419	FBgn0029639		2779214	2779944	**-1.10**	**8.08**	**5.92E-25**	**0.22**	**8.06**	**1.51E-01**
CG3526	FBgn0040355		2785995	2787916	-0.02	6.74	8.28E-01	**-0.06**	**6.59**	**5.71E-01**
CG3588	FBgn0025643		2813803	2818416	**0.04**	**8.86**	**6.84E-01**	**0.16**	**8.30**	**6.99E-02**
CG14424	FBgn0025644		2820082	2821202	0.04	6.06	6.40E-01	**0.01**	**6.12**	**9.17E-01**
CG32793	FBgn0052793		2823829	2826575	**-0.13**	**6.35**	**7.77E-02**	**-0.02**	**6.22**	**7.40E-01**
CG3592	FBgn0029642		2829203	2829952	**0.05**	**7.44**	**3.64E-01**	**0.40**	**7.25**	**9.45E-20**
CG3598	FBgn0025645		2831161	2831979	0.01	6.16	9.35E-01	**0.12**	**6.11**	**1.54E-01**
CG14420	FBgn0029643		2832889	2834574	0.09	6.15	4.12E-01	-0.01	6.15	9.33E-01
CG14421	FBgn0029644		2835515	2836303	-0.24	9.31	4.56E-05	-0.37	8.67	6.98E-13
CG14422	FBgn0029645		2837509	2838529	0.09	6.16	2.98E-01	-0.01	6.17	9.32E-01
CG14423	FBgn0029646		2839436	2840629	**0.01**	**6.14**	**9.32E-01**	**0.00**	**6.13**	**9.59E-01**
CG17959	FBgn0029647		2841138	2841584	**0.00**	**6.84**	**9.72E-01**	**-0.09**	**6.69**	**4.91E-01**
CG3603	FBgn0029648		2842069	2842983	**-0.07**	**7.68**	**5.62E-01**	-0.30	7.09	7.66E-03
CG3939	FBgn0040396		3069152	3070257	**0.23**	**8.85**	**6.61E-03**	0.10	8.46	1.38E-01

**In bold**, top 5% genes with highest ΔHP1; CG = gene ID; FB = FlyBase gene ID; M = change in expression level (log_2_); A = expression level.

To verify our microarray expression data we used quantitative RT-PCR (qRT-PCR) to determine the relative expression of several genes (Figure 4D). This confirmed that *white *is down-regulated in *w*^*m4e *^(*P *= 2*10^-4^, Student's t-test) but not in *w*^*m51b *^(*P *= 0.48). None of three other tested genes in X_Syx4-CG3603_, including *CG14419*, showed significant down-regulation in either *w*^*m4e *^or *w*^*m51b*^. Taken together, these results identify *white *as the gene with the most prominent response to heterochromatin, while other genes in the X_Syx4-CG3603 _region are not (or only marginally) affected, despite the fact that they show elevated HP1 levels.

### Chromatin accessibility changes in X_Syx4-CG3603_

**Figure 5:**
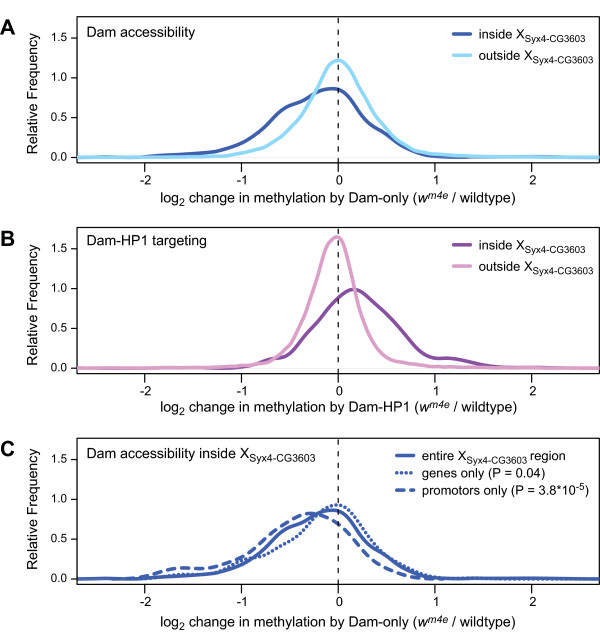
**Specific reduction of chromatin accessibility in X_Syx4-CG3603_**. A) Changes in chromatin accessibility determined from the Dam-only channel for all tiling array probes inside or outside the X_Syx4-CG3603 _region. Data are shown as 'density plots', which are smoothed histograms. B) Changes in methylation by Dam-HP1 fusion protein. C) Changes in chromatin accessibility in promoters (1 kb regions upstream of transcription start sites) and transcription units in the X_Syx4-CG3603 _region. *P*-value according to Wilcoxon rank test.

Heterochromatin formation is generally thought to lead to increased compaction of chromatin. Previously it was shown that upon heterochromatinization various genes in yeast and flies become less accessible to methylation by (unfused) Dam [48-51]. To study the effects of the *w*^*m *^rearrangements on chromatin accessibility, we analyzed the methylation levels as reported by the Dam-only channel of our DamID microarray data. Indeed, a majority of probes in the X_Syx4-CG3603 _region show a reduction in methylation by Dam-only in *w*^*m4e *^and *w*^*m51b *^flies compared with wild-type flies (Figure 5A and data not shown). This change is not found outside of the X_Syx4-CG3603 _region, which is consistent with the unaltered binding of HP1. In contrast to Dam-only, the Dam-HP1 methylation channel shows a specific enrichment in the X_Syx4-CG3603 _region (Figure 5B), due to targeting of the fusion protein to heterochromatin. These data show that the invasion of HP1 due to *w*^*m *^rearrangements is accompanied by a general decrease in chromatin accessibility.

Because the expression levels of all genes in X_Syx4-CG3603 _except *white *are not detectably altered in *w*^*m *^flies (Figure 4), we wondered whether the compaction of chromatin may be restricted to intergenic regions. Subsequent analysis revealed that promoters of the genes in X_Syx4-CG3603 _show a significant reduction in accessibility (Figure 5C). At the same time, the accessibility of the coding regions of these genes is not reduced. These results suggest that heterochromatinization differentially affects the accessibility of promoters and coding regions. The reduction in promoter accessibility at most genes in X_Syx4-CG3603 _is apparently not sufficient to cause detectable changes in gene expression, except in the case of *white*.

### HP1 binding to X_Syx4-CG3603 _is unusually sensitive to Su(var)3-9 dosage

**Figure 6:**
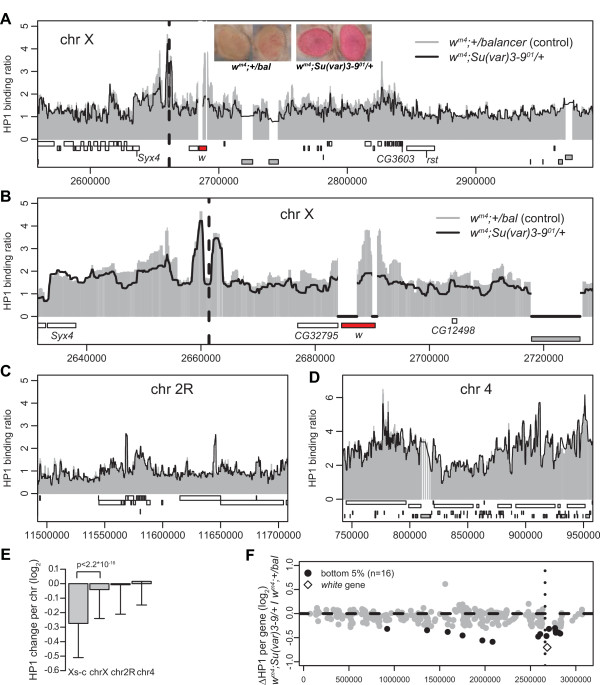
**Removal of one dose of *Su(var)3-9 *leads to subtle change of the HP1 binding pattern on *w*^*m4*^**. A) Chromosomal maps of HP1 binding in heterozygous *Su(var)3-9*^*01 *^and control flies. The *Su(var)3-9*^*01 *^allele is effectively a null allele [67]. HP1 binding profiles are shown for the same ~400 kb region as in Figure 1, E-G. Black dashed line indicates inversion breakpoint. Inset: eye color in *Su(var)3-9*^*01*^/+ and control male flies. B) Close up to ~90 kb region surrounding *white*; same region is plotted as in Figure 1H. C-D) Chromosomal maps of HP1 binding to parts of *chr 2R *and *chr 4*. E) Bar plot showing the average difference in HP1 binding (for each probe on the microarray averaged per chromosome) between control (*w*^*m4*^;+/*balancer*) and the mutant *w*^*m4*^; *Su(var)3-9*^*01*^/+. X_s-c _= X_Syx4-CG3603 _region; chrX = the first 3.2 Mb of the *chr X *excluding X_Syx4-CG3603_; chr2R = the parts of *chr 2R *that are covered on our array. Difference between X_S-C _and chrX, -0.27 log_2_; *P*-value < 2.2*10^-16^; Wilcoxon rank sum test. F) Chromosomal maps of first 3.4 Mb of X chr, with ΔHP1 (average change in log_2 _HP1 binding ratio) per gene. ΔHP1 between *w*^*m4*^; *Su(var)3-9*^*01*^/+ and control (*w*^*m4*^;+/*balancer*), Black dots show bottom 5% of genes (*n *= 16) for which ΔHP1 is largest. Black dotted line indicates inversion breakpoint.

*Su(var)3-9 *is one of the strongest modifiers of PEV known [52]. It was previously shown that the loss of a single allele of *Su(var)3-9 *dramatically decreases the silencing of reporter genes, including *white*, in a number of PEV reporter assays [53]. Indeed, heterozygous loss of *Su(var)3-9 *changes the eye color of *w*^*m4 *^males from nearly white to almost completely red (inset Figure 6A). This suggested that heterozygous loss of *Su(var)3-9 *may lead to destabilization of heterochromatin in the X_Syx4-CG3603 _region. To test this, we constructed HP1 binding maps in heads of mutant *w*^*m4*^*; Su(var)3-9*^*01*^/+ males, and of sibling *w*^*m4*^*;+/TM3, Sb Ser *control males. Flies carrying the TM3 balancer chromosome were used as a control because this balancer does not affect PEV [54-56]. The *w*^*m4 *^and *w*^*m4e *^X chromosomes originate from the same fly stock [34] and we confirmed that they have the same euchromatic breakpoint (see Methods). The binding maps of the control flies showed elevated HP1 levels in X_Syx4-CG3603 _on *w*^*m4*^, very similar to *w*^*m4e *^(Figure 6A and 6B). In contrast, heterozygous loss of *Su(var)3-9 *leads to a modest (log_2 _ratio -0.27, corresponding to ~0.8-fold) but statistically significant reduction of HP1 binding to X_Syx4-CG3603 _(Figure 6B and 6E), and especially to *white *(Figure 6B). Unlike in the X_Syx4-CG3603 _region, the HP1 levels on the probed heterochromatic segments of *chr 2R *and 4 are not affected by heterozygous loss of *Su(var)3-9 *(Figure 6C–E).

To systematically identify the genes on *chr X *that show the strongest reduction of HP1 after removal of one allele of *Su(var)3-9*, we calculated the change in HP1 binding per gene (Figure 6F) and selected the bottom 5% of genes (*n *= 16). Ten out of these 16 genes are located in the X_Syx4-CG3603 _region. Interestingly, these 10 genes are the same genes that gain HP1 on *w*^*m4e *^and *w*^*m51b*^. Thus, HP1 binding to the X_Syx4-CG3603 _region of *w*^*m4 *^is exceptionally sensitive to the levels of Su(var)3-9, whereas HP1 binding to other heterochromatic regions is more robust.

### Heterozygous loss of Su(var)3-9 specifically affects expression of white

**Figure 7:**
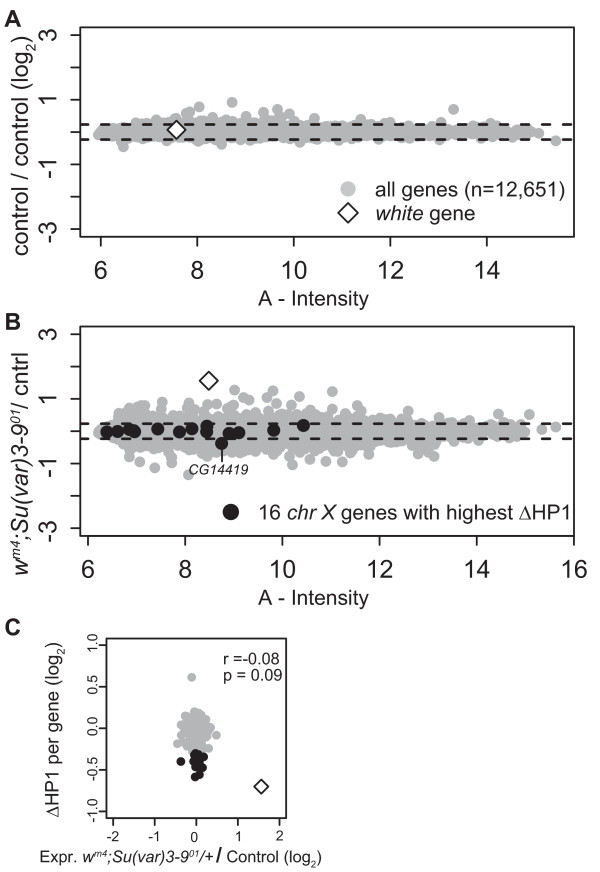
**No correlation between change in HP1 binding (ΔHP1) and change in gene expression**. A-B) MA-plots of expression profiles. Control (*w*^*m4*^;+/*balancer*) self-self (A) and *w*^*m4*^; *Su(var)3-9*^*01*^/+/control (B). Otherwise graphs have same layout and color usage as in Figure 4, A-C. C) Bivariate scatter plot of ΔHP1 against change in gene expression of *w*^*m4*^; *Su(var)3-9*^*01*^/+/control (*w*^*m4*^;+/*balancer*).

To examine the effect of heterozygous *Su(var)3-9 *loss on the expression of *white *and other genes, we made expression profiles in male heads from mutant *w*^*m4*^*; Su(var)3-9*^*01*^/+ and control *w*^*m4*^*;+/TM3, Sb Ser *flies (Figure 7A and 7B). The MA-plot of the mutant *w*^*m4*^*; Su(var)3-9*^*01*^/+ shows that only two of the 16 genes at which HP1 levels are most decreased (i.e. genes indicated with black dots in Figure 6F) also have an altered expression level (Figure 7B). These genes are *white*, which is expressed ~3-fold higher (log_2 _ratio = 1.56, *P *= 4.2*10^-41^), and *CG14419*, which is expressed slightly lower (log_2 _ratio = -0.38, *P *= 2.1*10^-8^). To confirm this result we repeated the microarray experiment using *w*^1118 ^flies, instead of *Oregon-R-S *as source of the wild-type Y chromosome and autosomes. The correlation between these two experiments was high (Spearman's rho = 0.54, *P *< 2.2*10^-16^, data not shown). We confirmed the up-regulation of *white *(log_2 _ratio = 1.28, *P *< 10^-45^), and found that *CG14419 *was slightly, but not significantly, down-regulated (log_2 _ratio = -0.16, *P *= 0.02).

Su(var)3-9 loss possibly affects HP1 binding and gene expression levels outside the X_Syx4-CG3603 _region. However, a bivariate scatterplot (Figure 7C) did not reveal any correlation between the changes in HP1 binding and the changes in expression level (Spearmans's rho = -0.08). This suggests that there is no association between HP1 loss and change in gene expression outside X_Syx4-CG3603_. Thus, in a *w*^*m4 *^background, loss of one allele of *Su(var)3-9 *mainly affects the expression level of *white*. None of the other genes in the X_Syx4-CG3603 _region are upregulated as a result of the reduced Su(var)3-9 dosage.

### Embryonic expression levels of genes in X_Syx4-CG3603 _do not explain the lack of repression in *w*^*m *^lines

**Figure 8:**
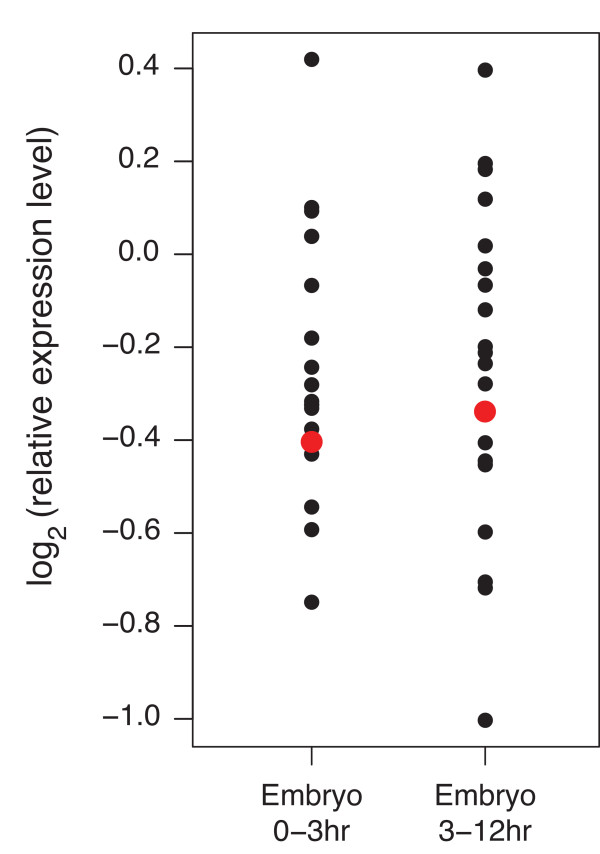
**Expression levels of all 20 genes in X_Syx4-CG3603 _during embryogenesis**. Expression data from wild-type embryos at two different stages are from [58]. Red dot represents the *white *gene.

It has been suggested that binding of transcriptional activators to the promoter can counteract heterochromatin-mediated silencing [57]. This protection was shown to depend on the level and timing of transcriptional activator expression. In particular, promoter activation in the embryonic stage could prevent silencing of a reporter later in development [57]. To investigate whether such a phenomenon may explain the preferential silencing of *white *by heterochromatin, we analyzed the embryonic expression levels [58] of all 20 genes in the X_Syx4-CG3603 _region (Figure 8). Several of these genes are expressed at even lower levels than *white*, suggesting that the absence of embryonic activity of *white *is not the sole determinant of its preferential silencing by heterochromatin.

## Discussion

The ability of heterochromatin to invade neighboring euchromatin was previously demonstrated by chromatin immunoprecipitation mapping studies in fission yeast (reviewed in [3,59]). In multicellular eukaryotes, HP1-containing chromatin is known to be able to associate with long stretches of DNA in a natural setting [17,19], but it has remained unclear how HP1 is redistributed in the context of PEV. In this study we have generated a high-resolution binding map of *Drosophila *HP1, a defining marker of heterochromatin, in the *white-mottled *PEV model. This model has been studied for more than 70 years but the underlying molecular events have remained enigmatic. On both *w*^*m4e *^and *w*^*m51b *^chromosomes we found that HP1 invades originally euchromatic regions to form new clearly defined heterochromatin domains. In total ~200 kb of DNA, including *white *and 19 other genes, is covered *de novo *by HP1. The pattern of HP1 fluctuations is highly reproducible between the *w*^*m4e *^and *w*^*m51b *^lines, and is therefore not due to random noise. A previous report found elevated H3K9me2 levels at selected loci on *w*^*m4 *^and proposed a gradient of heterochromatin from the breakpoint [46]. However, this study lacked the probing density required to determine the exact binding pattern, and our high-resolution map does not support a simple gradient model. A second study examined the *T(2;3)lt*^*x*13 ^reciprocal translocation and observed an overall enrichment of H3K9me2, with substantial local variation, extending ~200 kb across the breakpoint [60]. Our data show that also the absolute levels of HP1 binding in the newly formed heterochromatin domains display considerable local variation.

These results suggest an intermediate model between the previously proposed oozing and hopping models of PEV. Increased levels of HP1 are found along the entire X_Syx4-CG3603 _region, which is compatible with processive assembly of heterochromatin as in the oozing model. However, the variation in HP1 levels indicates that DNA sequence or epigenetic determinants locally modulate the efficiency of HP1 binding, as is inherent to the hopping model.

This variation of HP1 density along the chromosomal region is consistent with earlier observations. Discontinuities in the compaction of PEV regions have been observed at much lower resolution in polytene chromosomes [37], and examples have been reported of PEV in which a gene proximal to a heterochromatin block is transcribed while a more distal gene is silenced [34,35]. Hence, heterochromatin in PEV models can exhibit local differences in the level of HP1 binding and variation in the effect on gene expression.

A surprising result of our systematic study is that *white *is the only gene that is strongly repressed as a consequence of the invasion of heterochromatin. This finding is remarkable in the light of the important contributions of the *white-mottled *model to our understanding of heterochromatin. In hindsight, the behavior of *white *appears not to be prototypical of euchromatic genes that are brought into a heterochromatic environment. Genes may differ in their intrinsic response thresholds to heterochromatin levels [61], and *white *is perhaps particularly sensitive in this respect. The silencing of *white *by heterochromatin also contrasts with genome-wide mapping data that demonstrate that hundreds of genes are naturally bound by HP1, yet most of these are normally expressed [17], possibly because the binding levels of HP1 are below the threshold level that would cause their repression. Examples of genes that are bound and repressed by HP1 in their natural genomic context [21,62,63] are still rare. Some genes, particularly genes embedded in pericentric heterochromatin, may have evolved to become activated rather than repressed by heterochromatin, via a still unknown mechanism [64]. Thus, *white *may in fact be a 'red herring' that nevertheless has provided many insights into heterochromatin structure and function.

The variable effects on gene expression may be tightly linked to the local level of binding by heterochromatin proteins. Indeed, on *w*^*m4e*^, the *white *gene has the highest level of HP1 binding of all genes in X_Syx4-CG3603_, and *white *is also most strongly repressed. Slightly lower levels of HP1 on the *white *gene on *w*^*m51b*^, and the reduction in HP1 binding to *white *on *w*^*m4 *^upon loss of one *Su(var)3-9 *allele, both correlate with the restoration of *white *expression to near-wild-type levels. This all-or-none expression is a striking feature of PEV [65], and suggests that *white *has a threshold for heterochromatin mediated silencing. The other genes in the X_Syx4-CG3603 _region show typically lower levels of HP1 binding than *white*, and may not be silenced effectively because their threshold of epigenetic silencing is not reached.

HP1 binding to the X_Syx4-CG3603 _region is more sensitive to Su(var)3-9 dosage than regions that are naturally heterochromatic, such as *chr 4 *and pericentric heterochromatin of *chr 2R*. For stable heterochromatin formation, certain *cis*-acting elements may be necessary that might be lacking in the X_Syx4-CG3603 _region. Previous studies have indicated that repeat elements act cooperatively to stabilize heterochromatin [2,42,66]. The relatively low repeat content of X_Syx4-CG3603 _may explain the unusual sensitivity of HP1 binding in this region to the loss of one functional *Su(var)3-9 *allele. In addition, genes in this region may be relatively rich in binding sites for transcription regulators that counteract heterochromatin formation.

The epigenetic switching of *white*, resulting in all-or-none expression in the fly eye, can also be better understood by our observations. Our data indicate that the level of heterochromatin on *white *in *w*^*m4e *^is only a little bit above the silencing-threshold. Our data also show that small changes in heterochromatin levels especially affect the X_Syx4-CG3603 _region. As a result, subtle stochastic differences between cells, in for example the expression level of heterochromatin proteins, may especially change the transcriptional output of *white*. At the same time these small stochastic differences between cells will not affect the more stable natural heterochromatic regions.

It has been suggested that the range of heterochromatic spreading may be modulated by Su(var) proteins [46,67]. Our data indicate that Su(var)3-9 does not control the extent of spreading of HP1, because the borders of the *de novo *heterochromatin regions in *w*^*m4 *^do not shift upon the reduction of Su(var)3-9 dosage. Instead, we observe a general reduction of HP1 binding levels across most of the X_Syx4-CG3603 _region. This does not rule out that other Su(var) proteins may regulate the extent of spreading.

## Conclusion

Here we provide a detailed view of the linear organization of heterochromatin along the genomic regions involved in PEV. We find that HP1 invades euchromatin across the inversion breakpoints over ~175 kb and ~30 kb, causing *de novo *association of HP1 with 20 genes. The local variation of HP1 binding levels suggests an intricate interplay between heterochromatin proteins and local sequence context. The *white *gene has an unusual intrinsic affinity for heterochromatin, which may render this gene more easily silenced by heterochromatin than most other genes. Moreover, HP1 binding to the invaded region is exceptionally sensitive to the dosage of the histone methyltransferase Su(var)3-9, indicating that the *de novo *formed heterochromatin is less stable than most pericentric heterochromatin. Taken together, our molecular maps demonstrate that heterochromatin can invade a normally euchromatic region, yet the strength of HP1 binding and effects on gene expression are highly dependent on local context.

## Methods

### Fly stocks

*Oregon-R-S *(#4269) and *w*^*1118 *^(#3605) were obtained from Bloomington Drosophila Stock center. The *w*^*m4*^*;Su(var)3-9*^*01*^*/TM3, Sb Ser *stock, *In(1)w*^*m51*^,*w*^*m51b*^*ct *and *In(1)w*^*m4*^,*w*^*m4e *^(denoted as *w*^*m51b *^and *w*^*m4e*^, collectively referred to as *w*^*m*^) were kindly provided by P Talbert, Fred Hutchinson Cancer Research Center, Seattle, WA, USA. The inverted X chromosomes in these stocks are described in [34]. We examined the positions of the euchromatic inversion breakpoints of the *w*^*m4*^, *w*^*m51b *^and *w*^*m4e *^stocks by PCR (data not shown). The *w*^*m51b *^break is located between position 2,665,094 and 2,666,052 (R5), and the *w*^*m4 *^and *w*^*m4e *^breaks are both between position 2,661,012 and 2,662,278, which is ~1,000 bp more upstream than was previously thought [44]. This corresponds to ~(24.5–25.5) and ~(28.2–29.5) kb upstream of the beginning of the *white *gene (R5.4).

The Dam-HP1 transgenic line (*w*^*1118*^*;P{Dam-Myc-HP1,w*^+*mC*^*} HP23/TM6B*) was made in parallel with the Dam-HP1 transgenic line used in [42], but has not been published before. Dam-only transgenic lines (*w*^*1118*^*; P{Dam, w*^+*mC*^*} 1-1M/TM6B *and *w*^*1118*^*; P{Dam, w*^+*mC*^*} 1-4M/CyO*) were constructed for this study. The SacII/EcoRI fragment from pNDamMyc [68] encoding Myc tagged EcoDam was cloned into pUAST [69]. Germline transformations into *w*^*1118 *^were done by BestGene Inc, Chino Hills, CA, USA. Transformants were identified by their eye color and balanced. Dam and Dam-HP1 expression are driven from the un-induced, truncated heat-shock promoter in pUAST, yielding extremely low expression levels below the detection limit of Western blotting or immunofluorescence microscopy [68]. Thus, it is highly unlikely that the Dam-HP1 protein itself will alter heterochromatin structure.

### Fly crosses and culture conditions

Flies were raised at 25°C on standard cornmeal/molasses/agar medium. For DamID of HP1 we crossed *w*^*m51b*^, *w*^*m4e *^or *Oregon-R-S *virgin females to Dam (line 1-4M) and Dam-HP1 males. The heads of male progeny (expressing the Dam transgene and containing an inverted X chromosome) were removed using a razor blade, stored at -80°C and used for DamID. To map HP1 in presence of heterozygous *Su(var)3*-9^*01*^, we crossed *w*^*m4*^*;Su(var)3-9*^*01*^*/TM3, Sb Ser *virgin females to Dam (line 1-1M) and Dam-HP1 males, which both have the transgenes inserted in the 3^rd ^chr. Heads of male progeny expressing Dam or Dam-HP1 and heterozygous for *Su(var)3-9*^*01*^, and heads of males expressing Dam or Dam-HP1 and with balancer (*TM3, Sb Ser*) were collected. For expression profiling we crossed *w*^*m4*^*;Su(var)3-9*^*01*^*/TM3, Sb Ser *virgin females to *Oregon-R-S *or *w*^*1118 *^males. Heads of male progeny with and without *Su(var)3-9*^*01 *^were collected. Heads were collected < 25 hours after eclosion.

### Expression profiles

Total RNA was extracted from fly heads using Trizol (Invitrogen-Life Technologies). Labeling and hybridizations were done according to standard protocols [70] using printed oligonucleotide arrays [70]. Spot fluorescence ratios were normalized using a lowess fit per subarray [72]. Each set (e.g. *w*^*m4e *^vs. *Oregon-R-S*, or *w*^*m4*^*;Su(var)3-9*^*01*^*/+ vs. w*^*m4*^*;+/TM3, Sb Ser*) consisted of four hybridizations: two biological replicates were each done in a technical dye-swap fashion. Replicates were combined into weighted average ratio and confidence level (*P*-value) was calculated per gene using an error model [73], which was fine-tuned by self-self hybridizations.

### Quantitative RT-PCR

Total RNA was DNAse treated and reverse transcribed (Invitrogen, ThermoScript RT-PCR System for first-strand cDNA synthesis). qPCRs were run using TaqMan chemistry on a BioRad DNA Engine Peltier thermal cycler. Primers and probe sequences are available upon request. Expression levels of each gene were normalized to *Ide*, a housekeeping gene located on 3L. For each of the genotypes *w*^*m4e*^, *w*^*m51b *^and *Oregon-R-S *five RNA isolations (20 fly heads per isolation) were done, and normalized expression ratios were calculated for randomly selected *w*^*m*^/*Oregon-R-S *pairs. The resulting five ratios were averaged and are plotted in Figure 3D.

### DamID

DamID was done as described [42] with minor modifications. Detailed protocols are available at [74]. For each microarray hybridization the experimental and reference sample consisted of five heads each. Each experiment consisted of four hybridizations using biologically independent samples. 1 μg of amplified methylated DNA was labeled with ULS Cy-dyes (Kreatech, Amsterdam, The Netherlands). Dam-HP1 and Dam-only methylated DNA was co-hybridized in a two-color design on a custom-designed 4 × 44 K Agilent microarray. The Dam-HP1/Dam-only methylation ratio represents the level of HP1-targeted methylation, corrected for local differences in chromatin accessibility [41]. Probe sequences are based on the complete *Drosophila melanogaster *genome sequence, release 5, downloaded from [75] on March 7, 2007. Median probe spacing is 136 bp. Probe sequences do not contain GATC, the recognition sequence of Dam.

### Data analysis

All statistical analyses were performed in the R language and environment [76]. DamID data were normalized using R-packages limma [77] and vsn [78]. Raw data files were loaded in R and the weight of control spots was set to zero to exclude them from the results. We did not apply background correction to the data. Data was initially normalized between different arrays (method = 'vsn') and subsequently to the mean log_2 _binding ratio in the euchromatic part of 2R. Hence, a log-ratio of 0 corresponds to the mean HP1 binding level found in euchromatin. Because Dam and HP1-Dam transgenic flies were made using *mini-white *(*w*^+*mC*^) as a marker gene, all array probes overlapping with *mini*-*white *were excluded from analysis.

### Data availability

DamID and expression data are available from the Gene Expression Omnibus [79], accession GSE12395.

## Competing interests

The authors declare that they have no competing interests.

## Authors' contributions

MJV carried out DamID and expression profiling experiments, MJV, LP, and BvS performed data analysis. LP designed the microarray for DamID. MN and RMK performed all microarray hybridizations. MJV and WT conducted qRT-PCR experiments, and WT performed Western blotting. BvS and MJV conceived of the study, designed the experiments, and wrote the manuscript. All authors read and approved the final manuscript.
